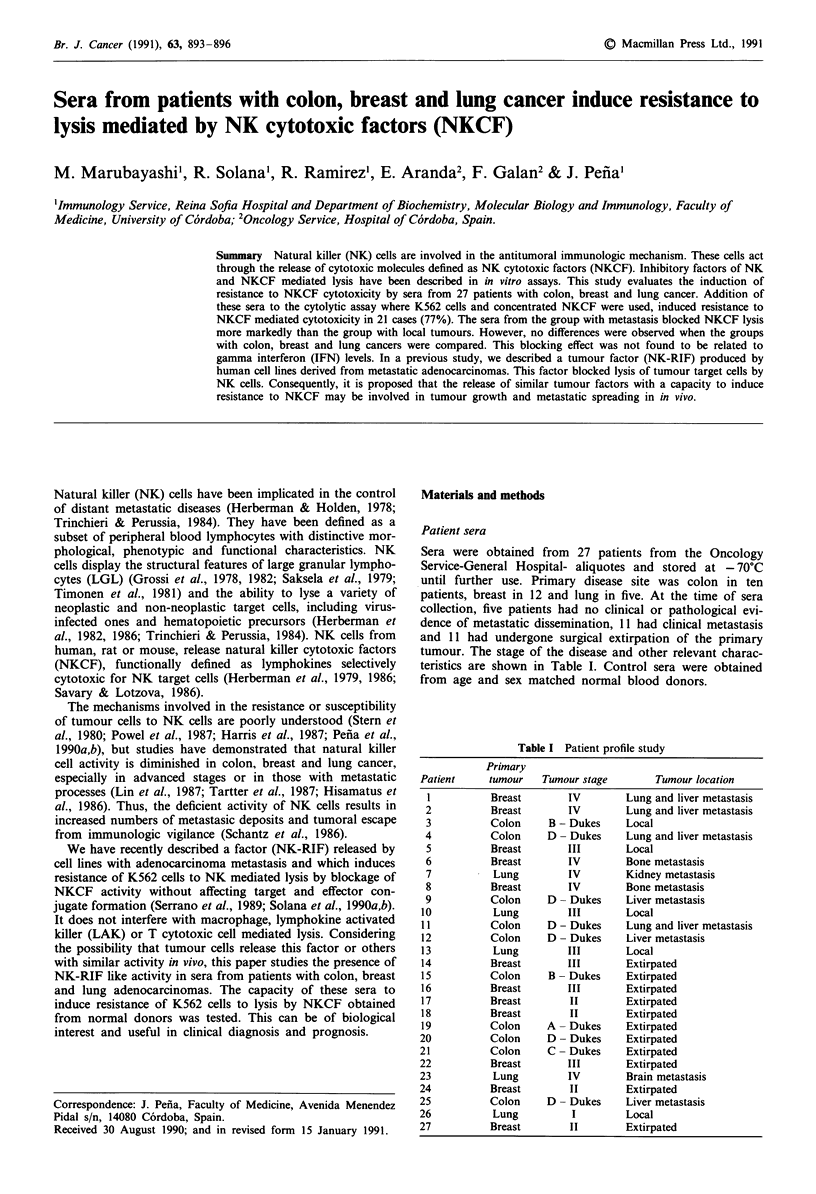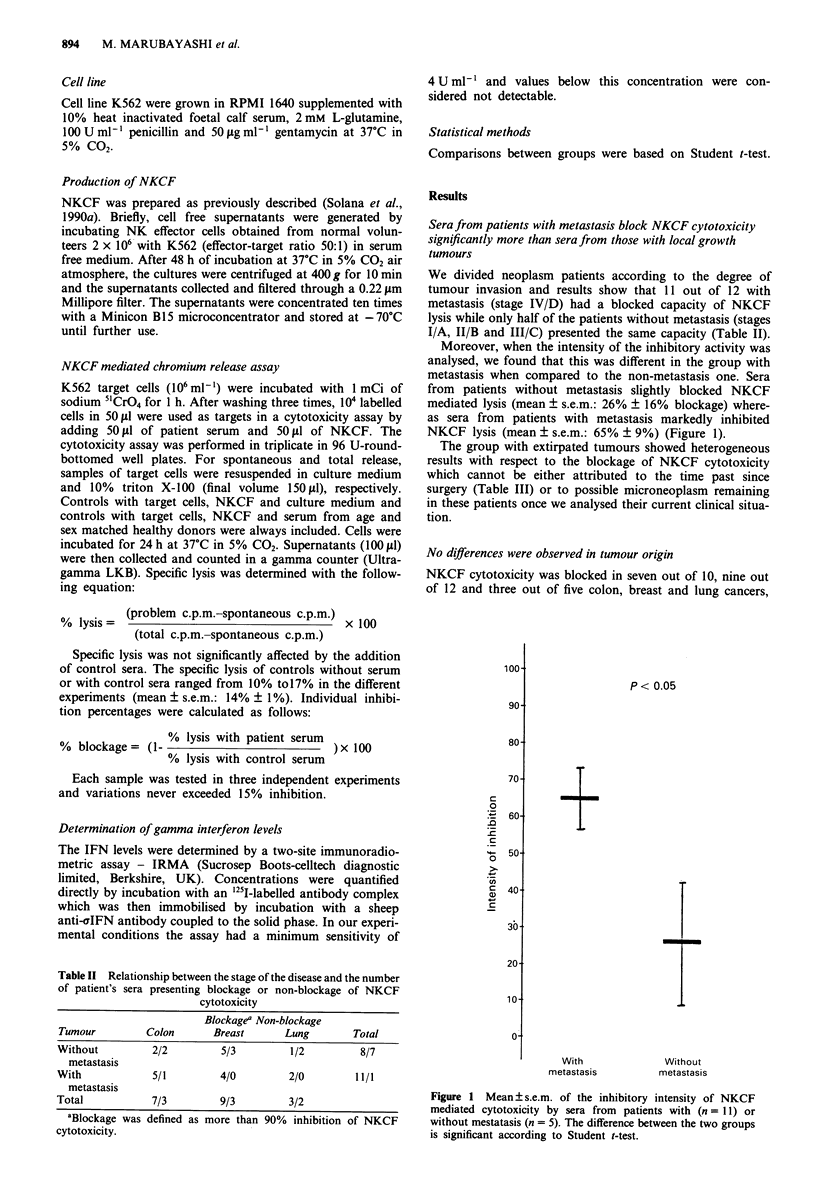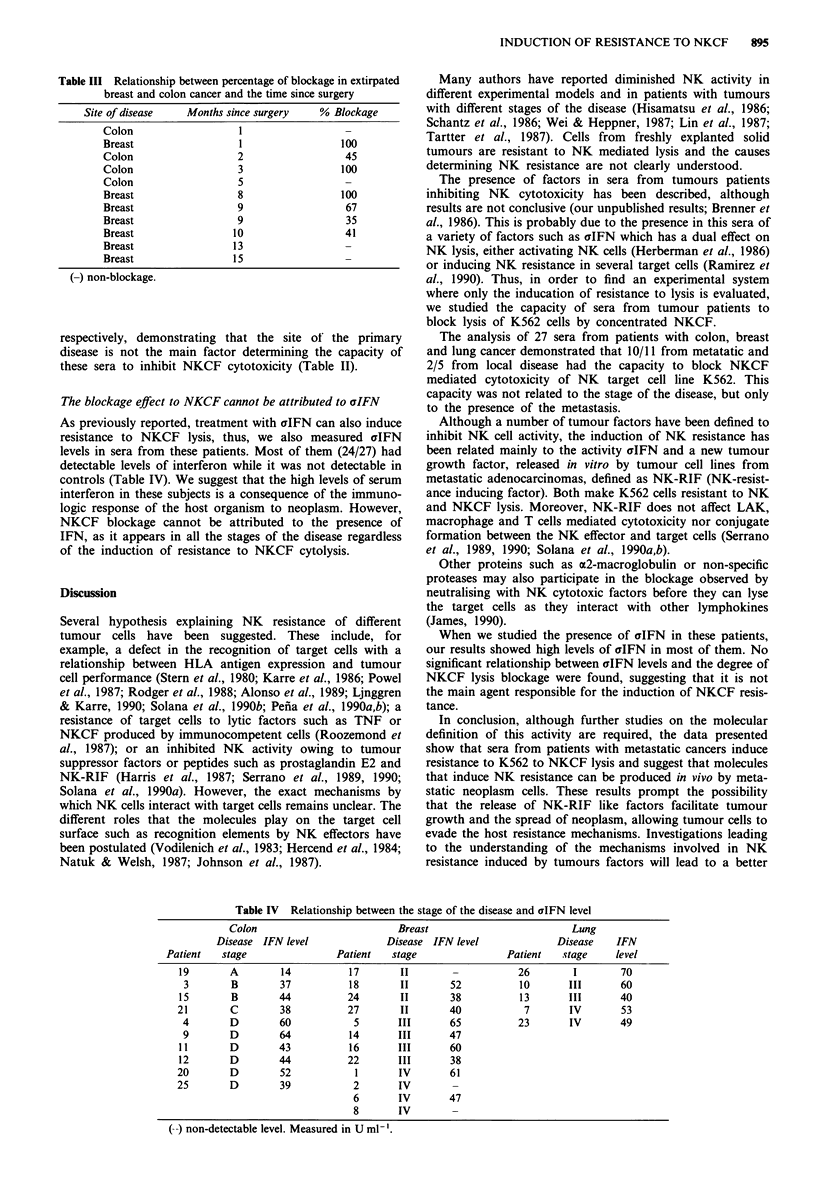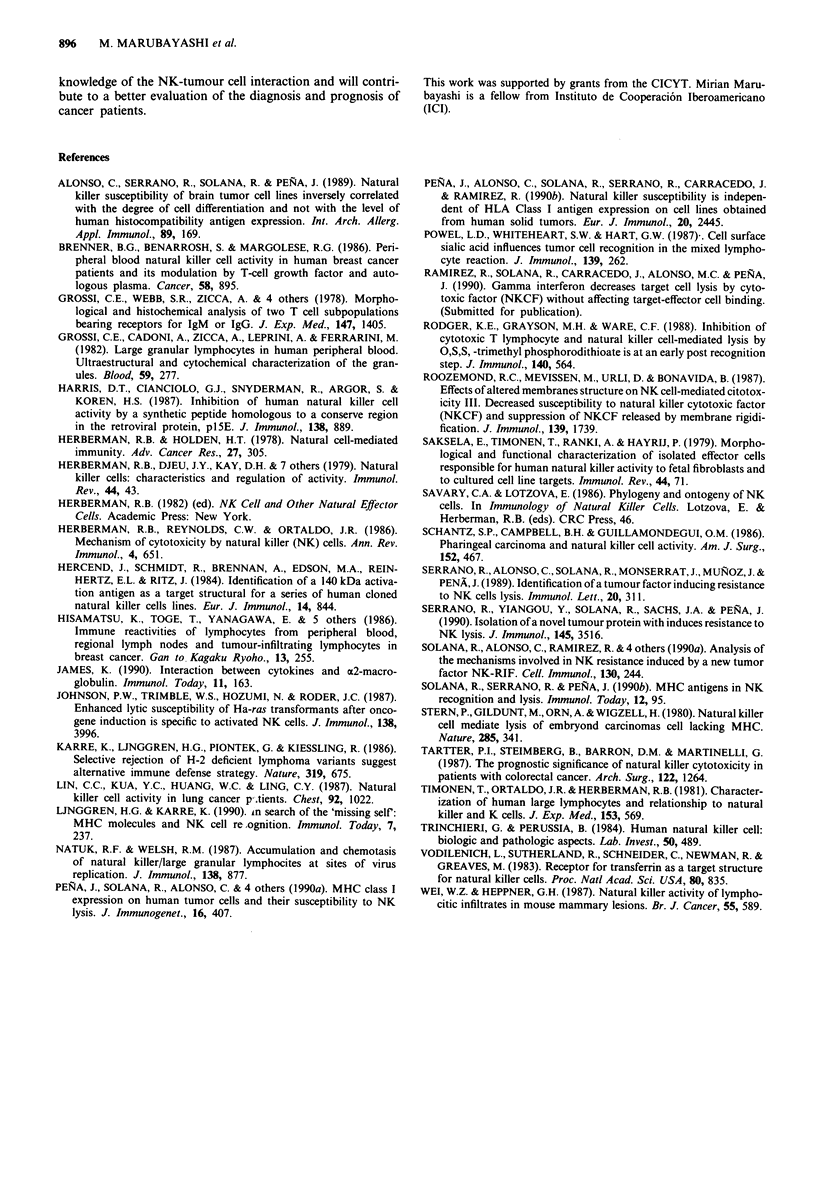# Sera from patients with colon, breast and lung cancer induce resistance to lysis mediated by NK cytotoxic factors (NKCF).

**DOI:** 10.1038/bjc.1991.196

**Published:** 1991-06

**Authors:** M. Marubayashi, R. Solana, R. Ramirez, E. Aranda, F. Galan, J. Peña

**Affiliations:** Immunology Service, Reina Sofia Hospital, Córdoba, Spain.

## Abstract

Natural killer (NK) cells are involved in the antitumoral immunologic mechanism. These cells act through the release of cytotoxic molecules defined as NK cytotoxic factors (NKCF). Inhibitory factors of NK and NKCF mediated lysis have been described in in vitro assays. This study evaluates the induction of resistance to NKCF cytotoxicity by sera from 27 patients with colon, breast and lung cancer. Addition of these sera to the cytolytic assay where K562 cells and concentrated NKCF were used, induced resistance to NKCF mediated cytotoxicity in 21 cases (77%). The sera from the group with metastasis blocked NKCF lysis more markedly than the group with local tumours. However, no differences were observed when the groups with colon, breast and lung cancers were compared. This blocking effect was not found to be related to gamma interferon (IFN) levels. In a previous study, we described a tumour factor (NK-RIF) produced by human cell lines derived from metastatic adenocarcinomas. This factor blocked lysis of tumour target cells by NK cells. Consequently, it is proposed that the release of similar tumour factors with a capacity to induce resistance to NKCF may be involved in tumour growth and metastatic spreading in in vivo.


*Br. J. Cancer* (1991), **63**, 893–896 © Macmillan Press Ltd., 1991

### Sera from patients with colon, breast and lung cancer induce resistance to lysis mediated by NK cytotoxic factors (NKCF)

M. Marubayashi[1], R. Solana[1], R. Ramirez[1], E. Aranda[2], F. Galan[2] & J. Peña[1]

[1]*Immunology Service, Reina Sofia Hospital and Department of Biochemistry, Molecular Biology and Immunology, Faculty of Medicine, University of Córdoba;* [2]*Oncology Service, Hospital of Córdoba, Spain.*

**Summary** Natural killer (NK) cells are involved in the antitumoral immunologic mechanism. These cells act through the release of cytotoxic molecules defined as NK cytotoxic factors (NKCF). Inhibitory factors of NK and NKCF mediated lysis have been described in *in vitro* assays. This study evaluates the induction of resistance to NKCF cytotoxicity by sera from 27 patients with colon, breast and lung cancer. Addition of these sera to the cytolytic assay where K562 cells and concentrated NKCF were used, induced resistance to NKCF mediated cytotoxicity in 21 cases (77%). The sera from the group with metastasis blocked NKCF lysis more markedly than the group with local tumours. However, no differences were observed when the groups with colon, breast and lung cancers were compared. This blocking effect was not found to be related to gamma interferon (IFN) levels. In a previous study, we described a tumour factor (NK-RIF) produced by human cell lines derived from metastatic adenocarcinomas. This factor blocked lysis of tumour target cells by NK cells. Consequently, it is proposed that the release of similar tumour factors with a capacity to induce resistance to NKCF may be involved in tumour growth and metastatic spreading in *in vivo*.

Natural killer (NK) cells have been implicated in the control of distant metastatic diseases (Herberman & Holden, 1978; Trinchieri & Perussia, 1984). They have been defined as a subset of peripheral blood lymphocytes with distinctive morphological, phenotypic and functional characteristics. NK cells display the structural features of large granular lymphocytes (LGL) (Grossi *et al.*, 1978, 1982; Saksela *et al.*, 1979; Timonen *et al.*, 1981) and the ability to lyse a variety of neoplastic and non-neoplastic target cells, including virus-infected ones and hematopoietic precursors (Herberman *et al.*, 1982, 1986; Trinchieri & Perussia, 1984). NK cells from human, rat or mouse, release natural killer cytotoxic factors (NKCF), functionally defined as lymphokines selectively cytotoxic for NK target cells (Herberman *et al.*, 1979, 1986; Savary & Lotzova, 1986).

The mechanisms involved in the resistance or susceptibility of tumour cells to NK cells are poorly understood (Stern *et al.*, 1980; Powel *et al.*, 1987; Harris *et al.*, 1987; Peña *et al.*, 1990*a,b*), but studies have demonstrated that natural killer cell activity is diminished in colon, breast and lung cancer, especially in advanced stages or in those with metastatic processes (Lin *et al.*, 1987; Tartter *et al.*, 1987; Hisamatus *et al.*, 1986). Thus, the deficient activity of NK cells results in increased numbers of metastasic deposits and tumoral escape from immunologic vigilance (Schantz *et al.*, 1986).

We have recently described a factor (NK-RIF) released by cell lines with adenocarcinoma metastasis and which induces resistance of K562 cells to NK mediated lysis by blockage of NKCF activity without affecting target and effector conjugate formation (Serrano *et al.*, 1989; Solana *et al.*, 1990*a,b*). It does not interfere with macrophage, lymphokine activated killer (LAK) or T cytotoxic cell mediated lysis. Considering the possibility that tumour cells release this factor or others with similar activity *in vivo*, this paper studies the presence of NK-RIF like activity in sera from patients with colon, breast and lung adenocarcinomas. The capacity of these sera to induce resistance of K562 cells to lysis by NKCF obtained from normal donors was tested. This can be of biological interest and useful in clinical diagnosis and prognosis.

Correspondence: J. Peña, Faculty of Medicine, Avenida Menendez Pidal s/n, 14080 Córdoba, Spain.
Received 30 August 1990; and in revised form 15 January 1991.

### Materials and methods

### *Patient sera*

Sera were obtained from 27 patients from the Oncology Service-General Hospital- aliquotes and stored at −70°C until further use. Primary disease site was colon in ten patients, breast in 12 and lung in five. At the time of sera collection, five patients had no clinical or pathological evidence of metastatic dissemination, 11 had clinical metastasis and 11 had undergone surgical extirpation of the primary tumour. The stage of the disease and other relevant characteristics are shown in Table I. Control sera were obtained from age and sex matched normal blood donors.

**Table I** Patient profile study

| *Patient* | *Primary tumour* | *Tumour stage* | *Tumour location* |
|---|---|---|---|
| 1 | Breast | IV | Lung and liver metastasis |
| 2 | Breast | IV | Lung and liver metastasis |
| 3 | Colon | B – Dukes | Local |
| 4 | Colon | D – Dukes | Lung and liver metastasis |
| 5 | Breast | III | Local |
| 6 | Breast | IV | Bone metastasis |
| 7 | Lung | IV | Kidney metastasis |
| 8 | Breast | IV | Bone metastasis |
| 9 | Colon | D – Dukes | Liver metastasis |
| 10 | Lung | III | Local |
| 11 | Colon | D – Dukes | Lung and liver metastasis |
| 12 | Colon | D – Dukes | Liver metastasis |
| 13 | Lung | III | Local |
| 14 | Breast | III | Extirpated |
| 15 | Colon | B – Dukes | Extirpated |
| 16 | Breast | III | Extirpated |
| 17 | Breast | II | Extirpated |
| 18 | Breast | II | Extirpated |
| 19 | Colon | A – Dukes | Extirpated |
| 20 | Colon | D – Dukes | Extirpated |
| 21 | Colon | C – Dukes | Extirpated |
| 22 | Breast | III | Extirpated |
| 23 | Lung | IV | Brain metastasis |
| 24 | Breast | II | Extirpated |
| 25 | Colon | D – Dukes | Liver metastasis |
| 26 | Lung | I | Local |
| 27 | Breast | II | Extirpated |

*Cell line*

Cell line K562 were grown in RPMI 1640 supplemented with 10% heat inactivated foetal calf serum, 2 mM L-glutamine, 100 U ml$^{-1}$ penicillin and 50 $\mu$g ml$^{-1}$ gentamycin at 37°C in 5% $CO_2$.

*Production of NKCF*

NKCF was prepared as previously described (Solana *et al.*, 1990*a*). Briefly, cell free supernatants were generated by incubating NK effector cells obtained from normal volunteers 2 × 10$^6$ with K562 (effector-target ratio 50:1) in serum free medium. After 48 h of incubation at 37°C in 5% $CO_2$ air atmosphere, the cultures were centrifuged at 400 *g* for 10 min and the supernatants collected and filtered through a 0.22 $\mu$m Millipore filter. The supernatants were concentrated ten times with a Minicon B15 microconcentrator and stored at −70°C until further use.

*NKCF mediated chromium release assay*

K562 target cells (10$^6$ ml$^{-1}$) were incubated with 1 mCi of sodium $^{51}CrO_4$ for 1 h. After washing three times, 10$^4$ labelled cells in 50 $\mu$l were used as targets in a cytotoxicity assay by adding 50 $\mu$l of patient serum and 50 $\mu$l of NKCF. The cytotoxicity assay was performed in triplicate in 96 U-round-bottomed well plates. For spontaneous and total release, samples of target cells were resuspended in culture medium and 10% triton X-100 (final volume 150 $\mu$l), respectively. Controls with target cells, NKCF and culture medium and controls with target cells, NKCF and serum from age and sex matched healthy donors were always included. Cells were incubated for 24 h at 37°C in 5% $CO_2$. Supernatants (100 $\mu$l) were then collected and counted in a gamma counter (Ultragamma LKB). Specific lysis was determined with the following equation:

$$\% \text{ lysis} = \frac{(\text{problem c.p.m.–spontaneous c.p.m.})}{(\text{total c.p.m.–spontaneous c.p.m.})} \times 100$$

Specific lysis was not significantly affected by the addition of control sera. The specific lysis of controls without serum or with control sera ranged from 10% to17% in the different experiments (mean ± s.e.m.: 14% ± 1%). Individual inhibition percentages were calculated as follows:

$$\% \text{ blockage} = (1 - \frac{\% \text{ lysis with patient serum}}{\% \text{ lysis with control serum}}) \times 100$$

Each sample was tested in three independent experiments and variations never exceeded 15% inhibition.

*Determination of gamma interferon levels*

The IFN levels were determined by a two-site immunoradiometric assay – IRMA (Sucrosep Boots-celltech diagnostic limited, Berkshire, UK). Concentrations were quantified directly by incubation with an $^{125}$I-labelled antibody complex which was then immobilised by incubation with a sheep anti-σIFN antibody coupled to the solid phase. In our experimental conditions the assay had a minimum sensitivity of 4 U ml$^{-1}$ and values below this concentration were considered not detectable.

*Statistical methods*

Comparisons between groups were based on Student *t*-test.

### Results

*Sera from patients with metastasis block NKCF cytotoxicity significantly more than sera from those with local growth tumours*

We divided neoplasm patients according to the degree of tumour invasion and results show that 11 out of 12 with metastasis (stage IV/D) had a blocked capacity of NKCF lysis while only half of the patients without metastasis (stages I/A, II/B and III/C) presented the same capacity (Table II).

Moreover, when the intensity of the inhibitory activity was analysed, we found that this was different in the group with metastasis when compared to the non-metastasis one. Sera from patients without metastasis slightly blocked NKCF mediated lysis (mean ± s.e.m.: 26% ± 16% blockage) whereas sera from patients with metastasis markedly inhibited NKCF lysis (mean ± s.e.m.: 65% ± 9%) (Figure 1).

The group with extirpated tumours showed heterogeneous results with respect to the blockage of NKCF cytotoxicity which cannot be either attributed to the time past since surgery (Table III) or to possible microneoplasm remaining in these patients once we analysed their current clinical situation.

*No differences were observed in tumour origin*

NKCF cytotoxicity was blocked in seven out of 10, nine out of 12 and three out of five colon, breast and lung cancers,

**Table II** Relationship between the stage of the disease and the number of patient's sera presenting blockage or non-blockage of NKCF cytotoxicity

| | | *Blockage*[a] *Non-blockage* | | |
|---|---|---|---|---|
| *Tumour* | *Colon* | *Breast* | *Lung* | *Total* |
| Without metastasis | 2/2 | 5/3 | 1/2 | 8/7 |
| With metastasis | 5/1 | 4/0 | 2/0 | 11/1 |
| Total | 7/3 | 9/3 | 3/2 | |

[a]Blockage was defined as more than 90% inhibition of NKCF cytotoxicity.

**Figure 1** Mean ± s.e.m. of the inhibitory intensity of NKCF mediated cytotoxicity by sera from patients with ($n = 11$) or without mestatasis ($n = 5$). The difference between the two groups is significant according to Student *t*-test.

**Table III** Relationship between percentage of blockage in extirpated breast and colon cancer and the time since surgery

| *Site of disease* | *Months since surgery* | *% Blockage* |
|---|---|---|
| Colon | 1 | – |
| Breast | 1 | 100 |
| Colon | 2 | 45 |
| Colon | 3 | 100 |
| Colon | 5 | – |
| Breast | 8 | 100 |
| Breast | 9 | 67 |
| Breast | 9 | 35 |
| Breast | 10 | 41 |
| Breast | 13 | – |
| Breast | 15 | – |

(–) non-blockage.

respectively, demonstrating that the site of the primary disease is not the main factor determining the capacity of these sera to inhibit NKCF cytotoxicity (Table II).

*The blockage effect to NKCF cannot be attributed to σIFN*

As previously reported, treatment with σIFN can also induce resistance to NKCF lysis, thus, we also measured σIFN levels in sera from these patients. Most of them (24/27) had detectable levels of interferon while it was not detectable in controls (Table IV). We suggest that the high levels of serum interferon in these subjects is a consequence of the immunologic response of the host organism to neoplasm. However, NKCF blockage cannot be attributed to the presence of IFN, as it appears in all the stages of the disease regardless of the induction of resistance to NKCF cytolysis.

### Discussion

Several hypothesis explaining NK resistance of different tumour cells have been suggested. These include, for example, a defect in the recognition of target cells with a relationship between HLA antigen expression and tumour cell performance (Stern *et al.*, 1980; Karre *et al.*, 1986; Powel *et al.*, 1987; Rodger *et al.*, 1988; Alonso *et al.*, 1989; Ljnggren & Karre, 1990; Solana *et al.*, 1990*b*; Peña *et al.*, 1990*a,b*); a resistance of target cells to lytic factors such as TNF or NKCF produced by immunocompetent cells (Roozemond *et al.*, 1987); or an inhibited NK activity owing to tumour suppressor factors or peptides such as prostaglandin E2 and NK-RIF (Harris *et al.*, 1987; Serrano *et al.*, 1989, 1990; Solana *et al.*, 1990*a*). However, the exact mechanisms by which NK cells interact with target cells remains unclear. The different roles that the molecules play on the target cell surface such as recognition elements by NK effectors have been postulated (Vodilenich *et al.*, 1983; Hercend *et al.*, 1984; Natuk & Welsh, 1987; Johnson *et al.*, 1987).

Many authors have reported diminished NK activity in different experimental models and in patients with tumours with different stages of the disease (Hisamatsu *et al.*, 1986; Schantz *et al.*, 1986; Wei & Heppner, 1987; Lin *et al.*, 1987; Tartter *et al.*, 1987). Cells from freshly explanted solid tumours are resistant to NK mediated lysis and the causes determining NK resistance are not clearly understood.

The presence of factors in sera from tumours patients inhibiting NK cytotoxicity has been described, although results are not conclusive (our unpublished results; Brenner *et al.*, 1986). This is probably due to the presence in this sera of a variety of factors such as σIFN which has a dual effect on NK lysis, either activating NK cells (Herberman *et al.*, 1986) or inducing NK resistance in several target cells (Ramirez *et al.*, 1990). Thus, in order to find an experimental system where only the inducation of resistance to lysis is evaluated, we studied the capacity of sera from tumour patients to block lysis of K562 cells by concentrated NKCF.

The analysis of 27 sera from patients with colon, breast and lung cancer demonstrated that 10/11 from metatatic and 2/5 from local disease had the capacity to block NKCF mediated cytotoxicity of NK target cell line K562. This capacity was not related to the stage of the disease, but only to the presence of the metastasis.

Although a number of tumour factors have been defined to inhibit NK cell activity, the induction of NK resistance has been related mainly to the activity σIFN and a new tumour growth factor, released *in vitro* by tumour cell lines from metastatic adenocarcinomas, defined as NK-RIF (NK-resistance inducing factor). Both make K562 cells resistant to NK and NKCF lysis. Moreover, NK-RIF does not affect LAK, macrophage and T cells mediated cytotoxicity nor conjugate formation between the NK effector and target cells (Serrano *et al.*, 1989, 1990; Solana *et al.*, 1990*a,b*).

Other proteins such as α2-macroglobulin or non-specific proteases may also participate in the blockage observed by neutralising with NK cytotoxic factors before they can lyse the target cells as they interact with other lymphokines (James, 1990).

When we studied the presence of σIFN in these patients, our results showed high levels of σIFN in most of them. No significant relationship between σIFN levels and the degree of NKCF lysis blockage were found, suggesting that it is not the main agent responsible for the induction of NKCF resistance.

In conclusion, although further studies on the molecular definition of this activity are required, the data presented show that sera from patients with metastatic cancers induce resistance to K562 to NKCF lysis and suggest that molecules that induce NK resistance can be produced *in vivo* by metastatic neoplasm cells. These results prompt the possibility that the release of NK-RIF like factors facilitate tumour growth and the spread of neoplasm, allowing tumour cells to evade the host resistance mechanisms. Investigations leading to the understanding of the mechanisms involved in NK resistance induced by tumours factors will lead to a better

**Table IV** Relationship between the stage of the disease and σIFN level

| | *Colon* | | | *Breast* | | | *Lung* | |
|---|---|---|---|---|---|---|---|---|
| *Patient* | *Disease stage* | *IFN level* | *Patient* | *Disease stage* | *IFN level* | *Patient* | *Disease stage* | *IFN level* |
| 19 | A | 14 | 17 | II | – | 26 | I | 70 |
| 3 | B | 37 | 18 | II | 52 | 10 | III | 60 |
| 15 | B | 44 | 24 | II | 38 | 13 | III | 40 |
| 21 | C | 38 | 27 | II | 40 | 7 | IV | 53 |
| 4 | D | 60 | 5 | III | 65 | 23 | IV | 49 |
| 9 | D | 64 | 14 | III | 47 | | | |
| 11 | D | 43 | 16 | III | 60 | | | |
| 12 | D | 44 | 22 | III | 38 | | | |
| 20 | D | 52 | 1 | IV | 61 | | | |
| 25 | D | 39 | 2 | IV | – | | | |
| | | | 6 | IV | 47 | | | |
| | | | 8 | IV | – | | | |

(–) non-detectable level. Measured in U $ml^{-1}$.

knowledge of the NK-tumour cell interaction and will contribute to a better evaluation of the diagnosis and prognosis of cancer patients.

This work was supported by grants from the CICYT. Mirian Marubayashi is a fellow from Instituto de Cooperación Iberoamericano (ICI).

### References

ALONSO, C., SERRANO, R., SOLANA, R. & PEÑA, J. (1989). Natural killer susceptibility of brain tumor cell lines inversely correlated with the degree of cell differentiation and not with the level of human histocompatibility antigen expression. *Int. Arch. Allerg. Appl. Immunol.*, **89**, 169.

BRENNER, B.G., BENARROSH, S. & MARGOLESE, R.G. (1986). Peripheral blood natural killer cell activity in human breast cancer patients and its modulation by T-cell growth factor and autologous plasma. *Cancer*, **58**, 895.

GROSSI, C.E., WEBB, S.R., ZICCA, A. & 4 others (1978). Morphological and histochemical analysis of two T cell subpopulations bearing receptors for IgM or IgG. *J. Exp. Med.*, **147**, 1405.

GROSSI, C.E., CADONI, A., ZICCA, A., LEPRINI, A. & FERRARINI, M. (1982). Large granular lymphocytes in human peripheral blood. Ultraestructural and cytochemical characterization of the granules. *Blood*, **59**, 277.

HARRIS, D.T., CIANCIOLO, G.J., SNYDERMAN, R., ARGOR, S. & KOREN, H.S. (1987). Inhibition of human natural killer cell activity by a synthetic peptide homologous to a conserve region in the retroviral protein, p15E. *J. Immunol.*, **138**, 889.

HERBERMAN, R.B. & HOLDEN, H.T. (1978). Natural cell-mediated immunity. *Adv. Cancer Res.*, **27**, 305.

HERBERMAN, R.B., DJEU, J.Y., KAY, D.H. & 7 others (1979). Natural killer cells: characteristics and regulation of activity. *Immunol. Rev.*, **44**, 43.

HERBERMAN, R.B. (1982) (ed). *NK Cell and Other Natural Effector Cells*. Academic Press: New York.

HERBERMAN, R.B., REYNOLDS, C.W. & ORTALDO, J.R. (1986). Mechanism of cytotoxicity by natural killer (NK) cells. *Ann. Rev. Immunol.*, **4**, 651.

HERCEND, J., SCHMIDT, R., BRENNAN, A., EDSON, M.A., REINHERTZ, E.L. & RITZ, J. (1984). Identification of a 140 kDa activation antigen as a target structural for a series of human cloned natural killer cells lines. *Eur. J. Immunol.*, **14**, 844.

HISAMATSU, K., TOGE, T., YANAGAWA, E. & 5 others (1986). Immune reactivities of lymphocytes from peripheral blood, regional lymph nodes and tumour-infiltrating lymphocytes in breast cancer. *Gan to Kagaku Ryoho.*, **13**, 255.

JAMES, K. (1990). Interaction between cytokines and α2-macroglobulin. *Immunol. Today*, **11**, 163.

JOHNSON, P.W., TRIMBLE, W.S., HOZUMI, N. & RODER, J.C. (1987). Enhanced lytic susceptibility of Ha-*ras* transformants after oncogene induction is specific to activated NK cells. *J. Immunol.*, **138**, 3996.

KARRE, K., LJNGGREN, H.G., PIONTEK, G. & KIESSLING, R. (1986). Selective rejection of H-2 deficient lymphoma variants suggest alternative immune defense strategy. *Nature*, **319**, 675.

LIN, C.C., KUA, Y.C., HUANG, W.C. & LING, C.Y. (1987). Natural killer cell activity in lung cancer p·tients. *Chest*, **92**, 1022.

LJNGGREN, H.G. & KARRE, K. (1990). in search of the 'missing self': MHC molecules and NK cell re·ognition. *Immunol. Today*, **7**, 237.

NATUK, R.F. & WELSH, R.M. (1987). Accumulation and chemotasis of natural killer/large granular lymphocites at sites of virus replication. *J. Immunol.*, **138**, 877.

PEÑA, J., SOLANA, R., ALONSO, C. & 4 others (1990*a*). MHC class I expression on human tumor cells and their susceptibility to NK lysis. *J. Immunogenet.*, **16**, 407.

PEÑA, J., ALONSO, C., SOLANA, R., SERRANO, R., CARRACEDO, J. & RAMIREZ, R. (1990*b*). Natural killer susceptibility is independent of HLA Class I antigen expression on cell lines obtained from human solid tumors. *Eur. J. Immunol.*, **20**, 2445.

POWEL, L.D., WHITEHEART, S.W. & HART, G.W. (1987)·. Cell surface sialic acid influences tumor cell recognition in the mixed lymphocyte reaction. *J. Immunol.*, **139**, 262.

RAMIREZ, R., SOLANA, R., CARRACEDO, J., ALONSO, M.C. & PEÑA, J. (1990). Gamma interferon decreases target cell lysis by cytotoxic factor (NKCF) without affecting target-effector cell binding. (Submitted for publication).

RODGER, K.E., GRAYSON, M.H. & WARE, C.F. (1988). Inhibition of cytotoxic T lymphocyte and natural killer cell-mediated lysis by O,S,S, -trimethyl phosphorodithioate is at an early post recognition step. *J. Immunol.*, **140**, 564.

ROOZEMOND, R.C., MEVISSEN, M., URLI, D. & BONAVIDA, B. (1987). Effects of altered membranes structure on NK cell-mediated citotoxicity III. Decreased susceptibility to natural killer cytotoxic factor (NKCF) and suppression of NKCF released by membrane rigidification. *J. Immunol.*, **139**, 1739.

SAKSELA, E., TIMONEN, T., RANKI, A. & HAYRIJ, P. (1979). Morphological and functional characterization of isolated effector cells responsible for human natural killer activity to fetal fibroblasts and to cultured cell line targets. *Immunol. Rev.*, **44**, 71.

SAVARY, C.A. & LOTZOVA, E. (1986). Phylogeny and ontogeny of NK cells. In *Immunology of Natural Killer Cells*. Lotzova, E. & Herberman, R.B. (eds). CRC Press, 46.

SCHANTZ, S.P., CAMPBELL, B.H. & GUILLAMONDEGUI, O.M. (1986). Pharingeal carcinoma and natural killer cell activity. *Am. J. Surg.*, **152**, 467.

SERRANO, R., ALONSO, C., SOLANA, R., MONSERRAT, J., MUÑOZ, J. & PEÑA, J. (1989). Identification of a tumour factor inducing resistance to NK cells lysis. *Immunol. Lett.*, **20**, 311.

SERRANO, R., YIANGOU, Y., SOLANA, R., SACHS, J.A. & PEÑA, J. (1990). Isolation of a novel tumour protein with induces resistance to NK lysis. *J. Immunol.*, **145**, 3516.

SOLANA, R., ALONSO, C., RAMIREZ, R. & 4 others (1990*a*). Analysis of the mechanisms involved in NK resistance induced by a new tumor factor NK-RIF. *Cell. Immunol.*, **130**, 244.

SOLANA, R., SERRANO, R. & PEÑA, J. (1990*b*). MHC antigens in NK recognition and lysis. *Immunol. Today*, **12**, 95.

STERN, P., GILDUNT, M., ORN, A. & WIGZELL, H. (1980). Natural killer cell mediate lysis of embryond carcinomas cell lacking MHC. *Nature*, **285**, 341.

TARTTER, P.I., STEIMBERG, B., BARRON, D.M. & MARTINELLI, G. (1987). The prognostic significance of natural killer cytotoxicity in patients with colorectal cancer. *Arch. Surg.*, **122**, 1264.

TIMONEN, T., ORTALDO, J.R. & HERBERMAN, R.B. (1981). Characterization of human large lymphocytes and relationship to natural killer and K cells. *J. Exp. Med.*, **153**, 569.

TRINCHIERI, G. & PERUSSIA, B. (1984). Human natural killer cell: biologic and pathologic aspects. *Lab. Invest.*, **50**, 489.

VODILENICH, L., SUTHERLAND, R., SCHNEIDER, C., NEWMAN, R. & GREAVES, M. (1983). Receptor for transferrin as a target structure for natural killer cells. *Proc. Natl Acad. Sci. USA*, **80**, 835.

WEI, W.Z. & HEPPNER, G.H. (1987). Natural killer activity of lymphocitic infiltrates in mouse mammary lesions. *Br. J. Cancer*, **55**, 589.